# Breast Cancer MDA-MB-231 Cells Use Secreted Heat Shock Protein-90alpha (Hsp90α) to Survive a Hostile Hypoxic Environment

**DOI:** 10.1038/srep20605

**Published:** 2016-02-05

**Authors:** Hangming Dong, Mengchen Zou, Ayesha Bhatia, Priyamvada Jayaprakash, Florence Hofman, Qilong Ying, Mei Chen, David T. Woodley, Wei Li

**Affiliations:** 1 Department of Dermatology and the Norris Comprehensive Cancer Centre, University of Southern California Keck Medical Centre,Los Angeles, CA 90033, USA; 2 Department of Pathology, University of Southern California Keck Medical Centre,Los Angeles, CA 90033, USA; 3 Eli and Edythe Broad Centre for Regenerative Medicine and Stem Cell Research and Department of Cell & Neurobiology, University of Southern California Keck Medical Centre, Los Angeles, CA 90033, USA

## Abstract

Rapidly growing tumours *in vivo* often outgrow their surrounding available blood supply, subjecting themselves to a severely hypoxic microenvironment. Understanding how tumour cells adapt themselves to survive hypoxia may help to develop new treatments of the tumours. Given the limited blood perfusion to the enlarging tumour, whatever factor(s) that allows the tumour cells to survive likely comes from the tumour cells themselves or its associated stromal cells. In this report, we show that HIF-1α-overexpressing breast cancer cells, MDA-MB-231, secrete heat shock protein-90alpha (Hsp90α) and use it to survive under hypoxia. Depletion of Hsp90α secretion from the tumour cells was permissive to cytotoxicity by hypoxia, whereas supplementation of Hsp90α-knockout tumour cells with recombinant Hsp90α, but not Hsp90β, protein prevented hypoxia-induced cell death via an autocrine mechanism through the LDL receptor-related protein-1 (LRP1) receptor. Finally, direct inhibition of the secreted Hsp90α with monoclonal antibody, 1G6-D7, enhanced tumour cell death under hypoxia. Therefore, secreted Hsp90α is a novel survival factor for certain tumours under hypoxia.

Hypoxia is a hallmark of solid tumours due to limited blood supply^1^. The protein level of the hypoxia-inducible factor-1alpha (HIF-1α) is a critical intracellular marker for sensing the environmental oxygen levels and a key regulator of cellular oxygen homeostasis in mammalian cells^2^. In normal cells, HIF-1α is low or undetectable under normal oxygen conditions (normoxia) and becomes accumulated in the cells when the oxygen levels drop to less than 2% (hypoxia). Among all the tumour samples screened, HIF-1α expression is found constitutive in approximately 50% of them due to activated oncogenes or deactivated tumour suppressor genes, regardless of the environmental oxygen content^2^,^3^. The high levels of HIF-1α in tumours, such as breast cancers, correlate with the large tumour size, high grade, high risk of metastasis and poor overall survival rate^4^,^5^. Therefore, inhibiting the constitutive HIF-1α function should slow down the progression of a wide variety of human tumours^1^,^2^,^3^. However, directly targeting the nucleus-located HIF-1 (α and β dimer) has proven to be challenging and so far few HIF-1 inhibitors have progressed through clinical development, raising the question of whether HIF-1 is a legitimate pharmacological target in those cancer patients^6^,^7^,^8^,^9^,^10^.

Like HIF-1α, the heat shock protein-90 (Hsp90) family members have been found either quantitatively over-expressed or qualitatively over-activated in a variety of tumours^11^,^12^,^13^,^14^. These either “extra” or “overactive” Hsp90 proteins are thought to act as chaperones to stabilize many oncoproteins inside the tumour cells and, therefore, have triggered excitement for development of Hsp90 inhibitors as anti-cancer therapeutics^11^,^12^,^15^. Geldanamycin (GM, or benzoquinone ansamycin) and its derivatives, such as 17-AAG (benzoquinone ansamycin 17-allylaminogeldanamycin) that inhibit the ATPase activity of Hsp90 proteins, entered numerous clinical trials since 1999^15^,^16^, but so far few have received approval for clinical applications. The small molecules’ instability and cytotoxicity remain among the hurdles.

Studies of the past decade, in particular, have uncovered a previously unrecognized location and function for Hsp90 family proteins, especially Hsp90α, its secreted form during tissue repair and cancer progression^17^,^18^,^19^,^20^. Similar to the regulation of HIF-1α, normal cells do not secrete Hsp90α unless under stress, such as tissue damage. In contrast, many tumours including skin, breast, colon, bladder, prostate, ovary, liver and bone, have been reported to constitutively secrete Hsp90α^20^. Down-regulation of HIF-1α or HIF-1β completely blocks Hsp90α secretion, indicating HIF-1 as a critical upstream regulator of Hsp90α secretion^19^,^21^. The best-characterized function for secreted Hsp90α is an unconventional pro-motility and pro-invasion factor, which acts via the cell surface receptor, LRP-1, as well as secreted MMP2 and other extracellular molecules^20^. Here we report a surprising finding that certain tumour cells secrete Hsp90α to protect themselves from hypoxia-triggered cell death.

## Results

**Figure 1:**
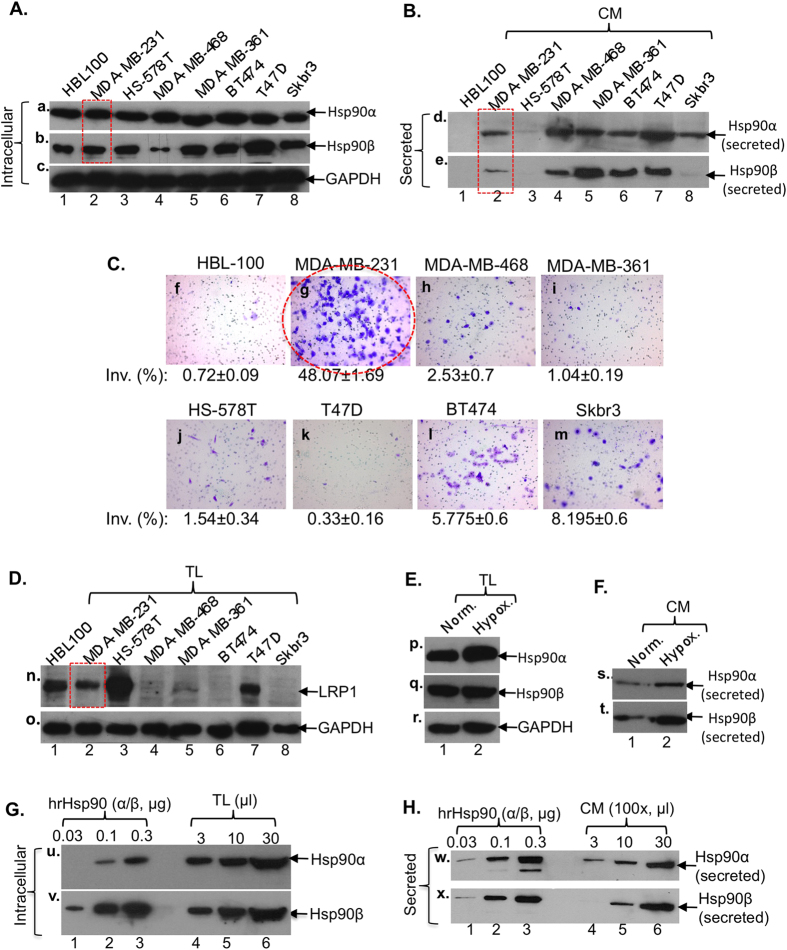
Selection of MDA-MB-231 breast cancer cell line as the model of study. Western blots for Hsp90α and Hsp90β in total lysates (TL) (**A**) or conditioned media (100×) (CM) (**B**) and the invasiveness (**C**) of the indicated breast cancer and control cell lines. Western blots for LRP-1 receptor among cell lines (**D**) and the total (**E**) and the secreted (**F**) Hsp90α and Hsp90β under hypoxia in MDA-MB-231. Intracellular (**G**) and secreted (**H**) ratios between Hsp90α and Hsp90β in MDA-MB-231 cells.

To choose a breast cancer cell model for study of the extracellular function of Hsp90α, we screened seven commonly used human breast cancer cell lines, with a non-transformed breast epithelial cell line as the control, for their expression and secretion of Hsp90α and Hsp90β. As shown in Fig. 1A, all cells expressed comparable amounts of Hsp90α (panel a) and Hsp90β (panel b) with an exception of MDA-MB-468 that showed a significantly lower expression of Hsp90β. Similarly, as shown in Fig. 1B, most of the cancer cells showed constitutive secretion of Hsp90α and Hsp90β, except Skbr3 that only secreted Hsp90α and HS-578T that showed no detectable secretion (panels d and e). As expected, like other normal cell types reported earlier, HBL-100 did not secrete either of the Hsp90 proteins under the similar conditions (lanes 1). Second, among the eight cell lines tested, MDA-MB-231 cells exhibited strong invasiveness in the Matrigel Invasion Assay (Fig. 1C, panel g), consistent with their original descriptions^22^. Third, interestingly, only three of the seven cancer cell lines express LRP1 (Fig. 1D, lanes 2, 3 and 7), a critical cell surface receptor for secreted Hsp90α-induced invasion *in vitro* and tumour formation in nude mice^21^,^23^,. The profile of LRP1 expression reflects the heterogeneity of human breast cancers. For instance, the HS-578T cells expressed the relatively highest level of LRP1 (lane 3), but did not secrete Hsp90 and could not invade. The reverse is true for MDA-MB-468 that lacks LRP1, showed poor invasion and could not form tumours in nude mice^21^. The T47D cells were an exception, which showed Hsp90α secretion and LRP1 expression, but much weaker invasion. It is possible that the LRP1B, an isoform and inhibitor of LRP1 function^24^, plays a dominant role over LRP1 in T47D cells.

Taking all the parameters into consideration, we chose the human triple negative breast cancer cell line, MDA-MB-231, as the cell model for this study. In these cells, acute hypoxia treatment (1% O2 for 6 hr) slightly elevated the amounts of intracellular (Fig. 1E, panel p) and secreted (Fig. 1F, panel s) Hsp90α, as well as Hsp90β (panel q and panel t), proteins. Through comparisons between intracellular Hsp90α (Fig. 1G, panel u, lanes 4–6) and Hsp90β (panel v, lanes 4–6) and between the secreted Hsp90α (Fig. 1H, panel w, lanes 4–6) and Hsp90β (panel x, lanes 4–6) with known amounts of human recombinant Hsp90α (panel w, lanes 1–3) and Hsp90β (panel x, lanes 1–3) by densitometry scanning, the amounts of the intracellular Hsp90α and Hsp90β were estimated as 7 ± 1.6 μg and 3 ± 0.7 μg present in 5 × 10^6^ MDA-MB-231 cells (~250 μg total cellular proteins) and the secreted Hsp90α and Hsp90β accounted for 7 ± 0.9% and 3 ± 0.5%, respectively, by the same number of cells. Thus, the ratio of both inside and outside Hsp90α versus Hsp90β is approximately 2:1 for MDA-MB-231 cells.

**Figure 2:**
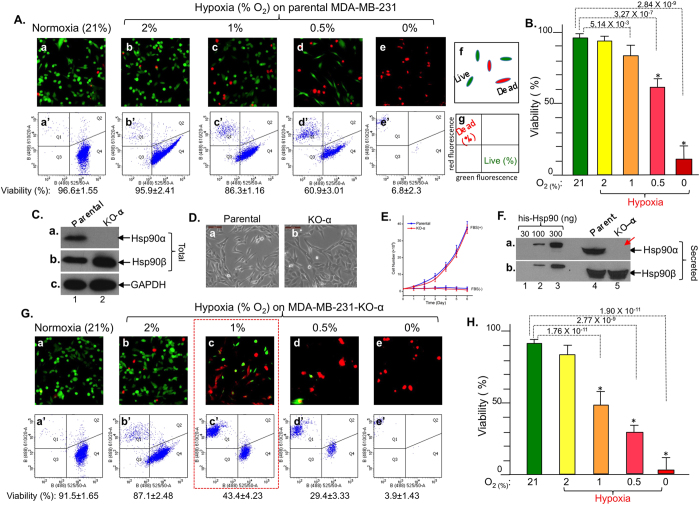
CRISPR-cas9 knockout of Hsp90α sensitizes MDA-MB-231 cells to hypoxia-driven killing. (**A**) Cell viability by fluorescence microscopy (panels a to e) and flow cytometry (panels a’ to e’). (**B**) Quantitation of viability data. (**C**) Evidence of CRISPR/Cas9 knockout of Hsp90α protein, (**D**) morphology and (**E**) proliferation profiles of parental and Hsp90α-knockout cells. (**F**) Depletion of Hsp90α (panel a), but not Hsp90β (panel b), secretion in Hsp90α-knockout cells. (**G**) Viability of Hsp90α-knockout cells under normoxia or various degrees of hypoxia (**H**) Quantitation of viability data. n = 3, *p < 0.05.

We examined the viability of MDA-MB-231 cells under either normoxia or a range (from 2% to 0% O_2_) of hypoxia, which uses the intrinsic apoptotic pathway to cause cell death^25^. As shown in Fig. 2A, results of the calcein AM (green) and ethidium homodimer-1 (red) double staining under fluorescence microscopy, as schematically depicted (panel f), showed that a majority of the cells survived under normoxia (panel a), 2% O_2_ (panel b) and 1% O_2_ (panel c) for 48 hours under serum-free conditions. However, a significant proportion of the cells (red) started to die when the oxygen content dropped below 0.5% (panels d and e). Flow cytometry analysis of the cells showed the percentage (%) of live versus dead cells (panels a’ to e’), as schematically depicted (panel g), consistent with the staining data under fluorescence microscopy. Quantitation of data based on multiple repeated flow cytometry experiments as cell viability is shown in Fig. 2B.

To investigate a possible role for secreted Hsp90α or Hsp90β in tumour cell survival under hypoxia, we created MDA-MB-231 cells in which Hsp90α or Hsp90β was depleted by using the CRISPR/Cas9 system. As shown in Fig. 2C, an isolated cell clone following drug selections (Methods) showed complete absence of Hsp90α protein (panel a, lane 2 vs. lane 1). In the same cells, Hsp90β was slightly elevated (panel b), consistent with previous reports of Hsp90α-RNAi-treated cells^20^,^21^. Hsp90α knockout did not affect the morphology (Fig. 2D, panel b vs. panel a) and proliferation profiles of the cells (Fig. 2E). Also, secreted Hsp90α was no longer detectable from conditioned medium of the Hsp90α-knockout cells (Fig. 2F, panel a, lane 5 vs. lane 4). As expected, Hsp90β secretion remained unaffected (panel b, lane 5 vs. lane 4). Interestingly, we were unable to obtain Hsp90β-knockout cell clones, suggesting that Hsp90β is essential for survival of tumour cells. These observations are consistent with previously reported findings that Hsp90β gene knockout is embryonic lethal in mice, whereas mice lacking Hsp90α develop normally^26^,^27^,^28^.

We therefore focused on the role of secreted Hsp90α in tumour cell survival under hypoxia. Intriguingly, we found significantly more death of the Hsp90α-knockout cells than their parental counterparts under hypoxia. As shown in Fig. 2G, calcein AM and ethidium homodimer-1 staining showed massive cell death (red) starting from hypoxia with 1% or lower oxygen content (panels c, d, e vs. panels a and b). Results of flow cytometry analysis of the cells confirmed the fluorescence microscopy data (panels c’, d’ e’ vs. panels a’ and b’). Quantitation of the data from three independent experiments is shown in Fig. 2H, which shows that approximately an additional 50% of Hsp90α-knockout cells did not survive under hypoxia. For rest of the experiments, we chose to use the condition of 1% oxygen under serum free conditions for 48 hours.

**Figure 3:**
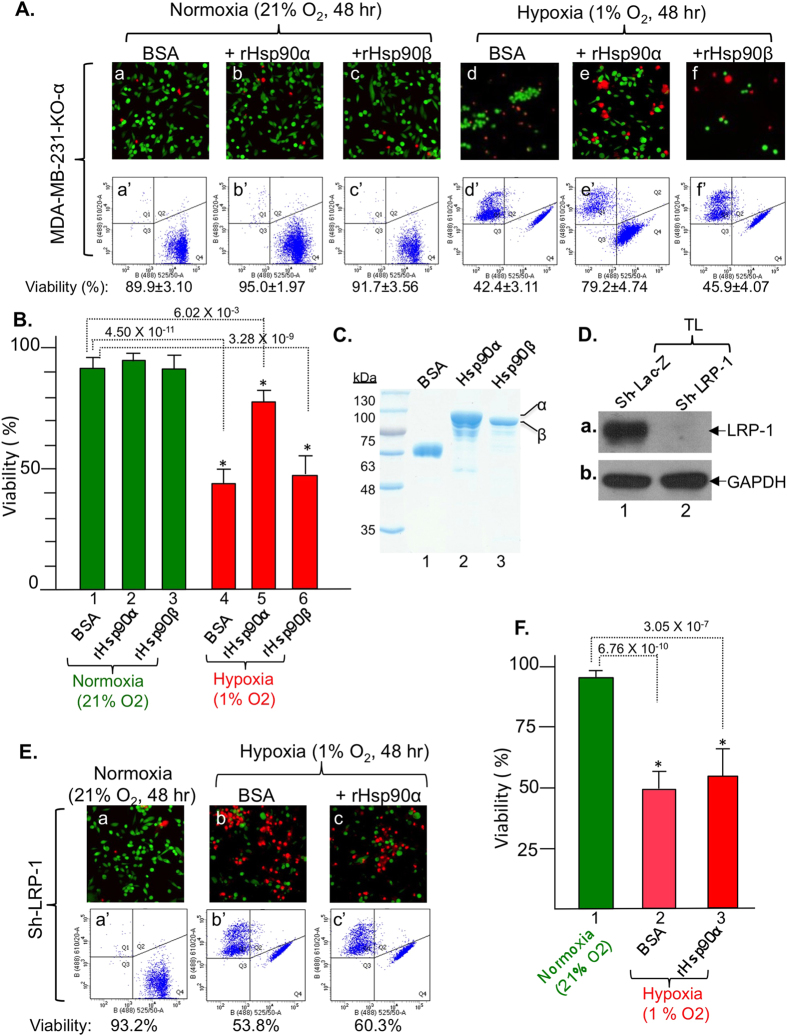
Rescue of Hsp90α-knockout cells from hypoxia-driven killing by extracellular Hsp90α, but not Hsp90β, protein via LRP-1 receptor signaling. (**A**) Extracellular Hsp90α, not Hsp90β, rescues viability of Hsp90α-knockout cells. (**B**) Quantitation of viability data. (**C**) Evidence of purified recombinant proteins for rescues. (**D**) Down-regulation of LRP-1 shown by Western blot. (**E**) No rescue of LRP-1-downregulated cells from hypoxia by extracellular Hsp90α. (**F**) Quantitation*. n* = 3, *p < 0.05.

Since Hsp90α gene knockout depletes both intracellular and extracellular Hsp90α, we tested whether it was the intracellular Hsp90α or the secreted Hsp90α that prevents cell death under hypoxia. We found that supplementing the Hsp90α-knockout cells with recombinant Hsp90α protein was sufficient to prevent hypoxia-induced cell death. As shown in Fig. 3A, the results of both fluorescent microscopy and flow cytometry assays of the stained cells showed that the majority (90%) of the Hsp90α-knockout cells survived under normoxia (panels a and a’). The addition of recombinant Hsp90α slightly improved the cell survival (panels b and b’), whereas recombinant Hsp90β did not show any effect (panels c and c’). However, more than 50% of the Hsp90α-knockout cells died under hypoxia (panels d and d’). Interestingly, supplementation of the Hsp90α-knockout cells with recombinant Hsp90α protein greatly prevented the hypoxia-induced cell death (panels e and e’). In comparison, recombinant Hsp90β protein did not show any rescue effects (panels f and f’). Quantitation of cell viability data is shown in Fig. 3B. The purified recombinant Hsp90α and Hsp90β proteins used for the rescue experiment were shown in Fig. 3C. Therefore the increased death of the Hsp90α-knockout tumour cells under hypoxia is likely due to lack of protection by secreted Hsp90α, but not Hsp90β.

We next tested if tumour-secreted Hsp90α utilizes an autocrine mechanism via the LRP1 receptor to protect the cells from hypoxia. This idea came from our previous reports that secreted Hsp90α binds to LRP1 to promote MDA-MB-231 cell invasion *in vitro* and tumour formation in nude mice^21^. In addition, Fuentealba *et al.* showed that LRP1-Akt signalling promotes neuronal cell survival^29^. As shown in Fig. 3D, down-regulation of LRP1 was nearly complete following infection with lentivirus carrying an shRNA against human LRP1 (panel a, lane 2 vs. lane 1). Hypoxia caused approximately 50% of the LRP1-downregulated cells to die (Fig. 3E, panels b and b’ vs. panels a and a’), similar to the death of the Hsp90α-knockout cells. Unlike Hsp90α-knockout cells, however, supplementation of the LRP1-downregulated cells with recombinant Hsp90α protein was unable to rescue the cells from hypoxia-driven killing (panels c and c’). Quantitation of the data is shown in Fig. 3F. We concluded that secreted Hsp90α promotes, via LRP1 receptor, tumour cell survival under hypoxia.

**Figure 4:**
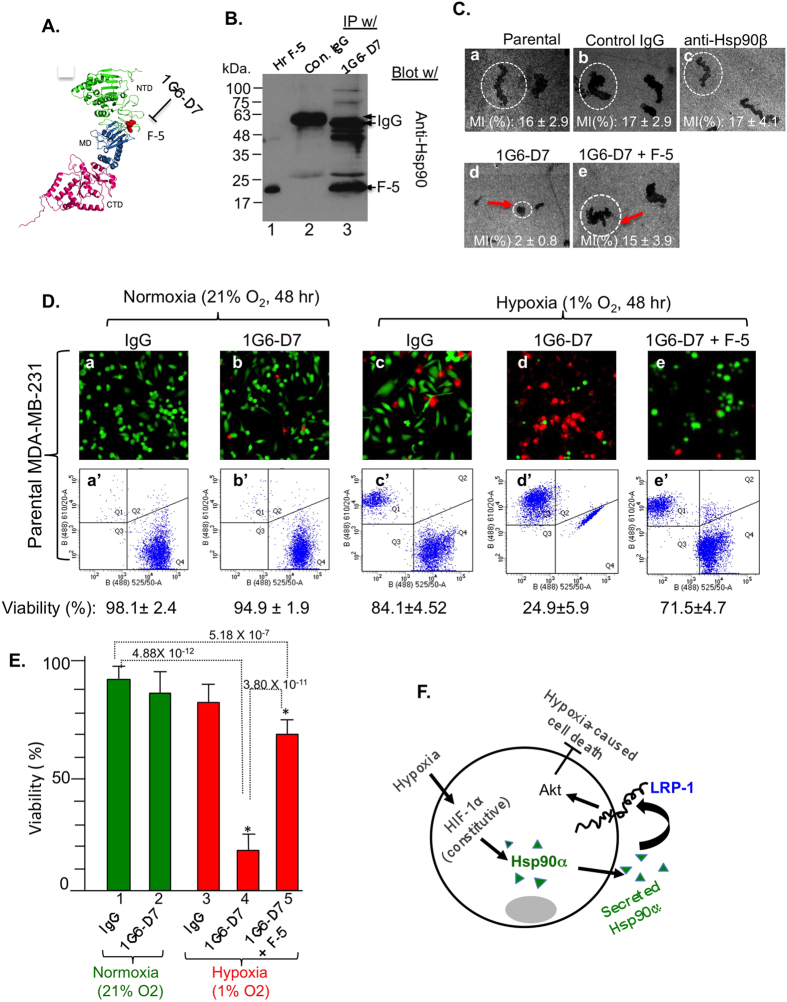
mAb 1G6-D7 neutralizes secreted Hsp90α and sensitizes MDA-MB-231 cells to hypoxia-driven killing. (**A**) A schematic location for the F-5 in Hsp90α and 1G6-D7’s recognition^21^,^25^. (**B**) mAb 1G6-D7 immunoprecipitates purified native F-5 protein. (**C**) mAb 1G6-D7 (10 μg/ml) blocked MDA-MB-231 cell migration (panel d) and F-5 (30 μg/ml) reversed the inhibition (panel e). (**D**) 1G6-D7 (panels d and d’) causes increased cell death under hypoxia and F-5 reversed 1G6-D7 effect. (**E**) Quantitation, n = 3, *p < 0.05. (**F**). A possible new mechanism for tumour cells’ survival under hypoxia: the HIF-1 > Hsp90α secretion > LRP1 receptor > Akt pathway.

To directly prove that it is the action of secreted Hsp90α that protects the tumour cells from hypoxia, we took approach of neutralization by a monoclonal antibody. We made use of a new monoclonal antibody recently developed in our laboratory, 1G6-D7, which recognizes the F-5 fragment of Hsp90α and strongly neutralizes the tumour-secreted Hsp90α function (Zou, M., Dong, H., Bhatia, A., Jayaprakash, P. and Li, W. unpublished). There is supporting evidence that a 115-amino acid fragment within the linker region and middle domain of Hsp90α, called F-5, retains the extracellular function of Hsp90α^29^. As schematically shown in Fig. 4A, 1G6-D7 binds to the F-5 region of Hsp90α, as evidenced by its capacity of immunoprecipitating the 19-kDa His-tagged F-5 protein, in comparison to non-specific mouse IgG (Fig. 4B, lane 3 vs. lane 2). More importantly, as shown in Fig. 4C, 1G6-D7 (panel d), but not the control mouse IgG (panel b) or anti-Hsp90β antibody (panel c), blocked MDA-MB-231 cell migration (panel a), which we have previously shown depends on secreted Hsp90α^21^. This inhibition was due to 1G6-D7 binding to F-5, since the addition of F-5 peptide reversed the inhibition (panel e). Under normoxia, 1G6-D7 showed little effect on the parental MDA-MB-231 cell survival, just like non-specific IgG (Fig. 4D, panels b and b’ vs. panels a and a’). Under hypoxia, cells with added control IgG showed 10–15% cell death (panels c and c’). However, the addition of 1G6-D7 caused approximately 75% cell death (panels d and d’ vs. panels c and c’). As expected, the addition of an excess amount of F-5 reversed the effect of 1G6-D7 (panels e and e’). Quantitation of the data, as shown in Fig. 4E, suggest that the tumour cells secrete Hsp90α to protect themselves from hypoxia-induced cell death. Finally, we compared four distinct human breast cancer or normal control cell lines, MDA-MB-231 (Hsp90α secretion^+^ and LRP1^+^), HBL-100 (Hsp90α secretion^−^ and LRP1^+^), HS-578T (Hsp90α secretion^−^ and LRP1^+^) and T47D (Hsp90α secretion^+^ and LRP1^+^). As shown in Supplementary figure 1 (Fig. 1s), we first down-regulated Hsp90α expression in these cells (A) using lentivirus carrying an shRNA as previously shown. As expected, hypoxia-caused death of MDA-MB-231 cells was rescued by recombinant Hsp90α protein. To our surprise, the addition of recombinant Hsp90α could not rescue hypoxia-caused death of other three cell lines included. While larger groups of cell lines would need to be tested to gain statistically meaningful conclusions, these data indicated that human breast cancers are highly heterogeneous and may use either different or more than one mechanism to survive under hypoxia.

## Discussion

Constitutive secretion of Hsp90, especially Hsp90α, has been reported for more than a dozen different tumour cell lines, in which a well characterized upstream regulator of secretion is HIF-1α^20^. A main reported function for tumour cell-secreted Hsp90α is to promote tumour cell motility and invasion. In this study, we have provided strong evidence to show that certain tumour cells, such as breast cancer cell line MDA-MB-231, secrete Hsp90α for another previously unrecognized purpose – to protect the tumour cells from hypoxia-triggered cell death. First, CRISPR/Cas9 gene knockout of Hsp90α caused more cell death than their parental counterparts under hypoxia. Second, supplementation with recombinant Hsp90α protein prevented the increased death of Hsp90α-knockout cells under hypoxia. Finally, a neutralizing monoclonal antibody against tumour cell-secreted Hsp90α caused more death of parental MDA-MB-231 cells under hypoxia. Together, we propose that secretion of Hsp90α represents a novel mechanism by which tumour cells survive the hostile hypoxic microenvironment. The same tumour cells also secrete Hsp90β, but the secreted Hsp90β was neither able to protect the cells from hypoxia-induced cell death nor able to compensate for the absence of secreted Hsp90α.

Hypoxia can cause cell death by necrosis or apoptosis or both. As a result of hypoxia, ATP levels drop and cellular activities in general cannot be maintained. If this stress lasts long enough, cells die. The hypoxia-caused cell necrosis is less understood. Hypoxia-caused cell apoptosis uses the intrinsic apoptotic pathway, involving HIF-1α^25^,^30^. However, previous studies reported opposite observations in either inducing or antagonizing apoptosis by accumulation of HIF-1α in the cells. First, induction of HIF-1α causes down-regulation of Bcl-2, an anti-apoptotic protein, and the up-regulation (via a HIF-1-responsive element, HRE) of the gene encoding Nip3, a pro-apoptotic member of cell death factors. Secondly, hypoxia activates the tumour suppressor gene p53 via a direct protein-protein interaction between HIF-1α and p53. This interaction leads to expression of a number of p53 target genes, such as the pro-apoptotic genes, Puma and Noxa, to initiate the apoptotic process^30^. On the other hand, a large number of studies showed that HIF-1α can protect cells from hypoxia-caused apoptosis. This discrepancy is likely due to differential contributions among HIF-1α, HIF-2α and HIF-3α, as well as the degree and duration of hypoxia^31^,^32^. The fact that secreted Hsp90α protects tumour cells from apoptosis supports the notion that HIF-1α antagonizes the apoptotic signals from hypoxia. How secreted Hsp90α prevents tumour cells from undergoing hypoxia-triggered cell death remains to be further established. In this study, we demonstrated the important role for the LRP1 receptor, which acts as a receptor for secreted Hsp90α and activates two major intracellular signalling pathways, the ERK1/2 and the Akt pathways^33^. Fuentealba and colleagues showed that the LRP1-Akt pathway promotes anti-apoptotic signalling in neurons^29^. Based on the known anti-apoptotic function of PI-3K/Akt in cell survival^34^, we propose that the tumour cells secrete Hsp90α and utilize the “secreted Hsp90α – LRP1 – Akt” autocrine circuit to survive hypoxia. This working model is schematically depicted in Fig. 4F.

Breast cancers are highly heterogeneous genetically, phenotypically and functionally and, therefore, there is no common treatment for different breast cancers in humans. Similarly, we do not believe that every type of breast cancer cells use secreted Hsp90α to survive hypoxia. For instance, HIF-1α is a central regulator of Hsp90α secretion in both normal and tumour cells^14^. However, only a fraction of solid tumours tested showed constitutively expressed HIF-1α^6^. Taking breast cancer again as an example, Dales and colleagues carried out anti-HIF-1α immunohistochemistry on frozen sections of 745 breast cancer samples and found that approximately 25–40% of all invasive breast cancer samples are hypoxic^4^. Unfortunately, directly targeting HIF-1α has proven not to be a viable approach for anti-tumour therapeutics^2^,^6^. We would like to argue that patients with HIF-1α overexpressing cancer and a high plasma level of Hsp90α could benefit from treatments such as the monoclonal antibody 1G6-D7, that selectively targets the secreted Hsp90α - a critical downstream effector of HIF-1α.

## Methods

### Cell lines

Eight human breast cancer cell and a control (untransformed) mammary epithelial cell lines were gifts from Dr. Michael Press (University of Southern California, Los Angeles). All the cells were cultured in DMEM medium supplemented with 10% fetal bovine serum (FBS), as well as ATCC-suggested media for some of the cell lines, such as McCoy’s 5A for Skbr3. Prior to experiments, the cells were deprived of serum and incubated under serum-free conditions for 16 hours. These cells were then subjected to designed experiments under normoxia or hypoxia.

### Hypoxia

Multi-chamber OxyCycler C42 from BioSpherix, Ltd (Redfield, NY) was used as the oxygen content controller in this study. This equipment allows creation of a full range of oxygen content regulation from 0.1% to 99.9%, as well as CO_2_ control from 0.1% to 20.0%. All media used in hypoxia experiments were pre-incubated in the chambers with the designated oxygen content overnight, as previously described^19^,^21^.

### Antibodies

Anti-Hsp90α specific antibody (CA1023) was obtained from Calbiochem (Billerica, MA) and anti-Hsp90β specific antibody (SMC 107) was from Stressmarq Biosciences (Victoria, BC, Canada). Anti-LRP1/CD91 antibody (37–7600) was purchased from Life Technologies (Grand Island, NY). Anti-GAPDH antibody (GTX28245) antibody was from Genetex (Irvine, CA).

### CRISPR-Cas9 knockout of Hsp90α and Hsp90β genes

We utilized the guide RNA (gRNA) synthesis protocol^37^ to identify all 23 bp genomic sites according to the form 5′-N_20_NGG-3′ near the target site of human Hsp90α gene (Gene ID:3320) and human Hsp90β gene (Gene ID:3326) and selected the following genomic site: 5′-GACCCAAGACCAACCGATGGAGG-3′ (Hsp90α) or 5′-GCTGATCTCATAAATAATTTGGG-3′ (Hsp90β) for synthesizing the gRNA. Then, the 5′-20 bp of the selected target sequence, i.e. 5′-GACCCAAGACCAACCGATGG-3′ (Hsp90α) or 5′-GCTGATCTCA TAAATAATTT-3′ (Hsp90β) was incorporated (underlined) into a 455 bp DNA fragment that bears all components necessary for gRNA expression, i.e. a U6 promoter + target sequence + guide RNA scaffold + termination signal as follows: TGTACAAAAAAGCAGGCTTTAAAGGAACCAATTCAGTCGACTGGATCCGGTACCAAGGTCGGGCAGGAAGAGGGCCTATTTCCCATGATTCCTTCATATTTGCATATACGATACAAGGCTGTTAGAGAGATAATTAGAATTAATTTGACTGTAAACACAAAGATATTAGTACAAAATACGTGACGTAGAAAGTAATAATTTCTTGGGTAGTTTGCAGTTTTAAAATTATGTTTTAAAATGGACTATCATATGCTTACCGTAACTTGAAAGTATTTCGATTTCTTGGCTTTATATATCTTGTGGAAAGGACGAAACACCGACCCAAGACCAACCGATGG(Hsp90α)orGCTGATCTCATAAATAATTT (Hsp90β)GTTTTAGAGCTAGAAATAGCAAGTTAAAATAAGGCTAGTCCGTTATCAACTTGAAAAAGTGGCACCGAGTCGGTGCTTTTTTTCTAGACCCAGCTTTCTTGTACAAAGTTGGCATTA. The entire DNA fragment was synthesized as guide Block (gBlock) by Integrated DNA Technologies, Inc. (Coraville, IA). For construction of the gRNA plasmid, the gBlock was amplified by PCR using primers (gRNA-block-EcoRI (F): GCGGAATTCTGTACAAAAAAGCAGGC and gRNA-block-EcoRI (R): GCGGAATTCTAATGCCAACTTTGTACA). The PCR amplicons were purified and subjected to EcoRI digestion and sub-cloned into the PiggyBac vector using the EcoRI site on the vector.

Approximately 1 × 10^6^ MDA-MB-231 cells were plated into each well of a 6-well plate, transfected with the gRNA and hCas9 plasmids using Lipofectamine® LTX & Plus Reagent (Life Technologies, Grand Island, NY). Twenty-four hours following transfection, the medium was replaced with fresh medium containing 10 μg/ml BSD and 700 μgml G418 and incubated for an additional 4 days with daily monitoring. Drug-resistant clones were isolated following drug selection using the “ring cloning” technique and the cloned cells plated into 60-mm tissue culture dishes. The levels of Hsp90 family proteins in the cells were analysed by Western blot analysis of cell extracts.

### Lentiviral systems for up- or down- regulation of target genes

The pRRLsinh-CMV system was used to overexpress exogenous genes. The FG-12 delivery system was used to deliver shRNAs against human LRP1 gene and Hsp90α gene, as previously described^33^,^35^,^36^.

### Monoclonal antibody, 1G6-D7, production

Monoclonal antibody against the F-5 fragment of Hsp90α, 1G6-D7, was developed in our laboratory. The immunogen preparation, immunization, screening and antibody epitope mapping are described in detail elsewhere (Zou, M., Dong, H., Bhatia, A., Jayaprakash, P. and Li, W., unpublished).

### Recombinant Hsp90α and Hsp90β production and purification

See details as previously described^35^.

### Preparation of serum-free conditioned medium

The detailed protocols for culturing cells, changing medium, incubation times, collecting serum-free conditioned medium, concentrating and analysing it by Western immunoblotting assays were as described previously^19^,^34^.

### Invasion assay

We followed the procedures as described by the manufacturer’s instruction (BD Biosciences, Bedford, MA). The Corning Biocoat Matrigel Invasion Chamber (Cat#354480) was used as detailed previously^21^. The invasion was calculated as the percentage (%) of the number of penetrated cells divided by the total number of cells plated.

### Cell viability assays

We used the LIVE/DEAD Viability/Cytotoxicity Kit for mammalian cells (MP 03224, Molecular Probes) and followed its (two) protocols: the Fluorescence Microscopy Protocol and the Flow Cytometry Protocol. Samples were analysed in triplicate for each condition.

### Statistical analyses

Data are based on three or four independent experiments. The data are presented as mean ± s.d. Matrigel Invasion Assay quantification was achieved by measuring five randomly selected fields per experimental condition. Colloidal gold salt migration assay quantification was achieved by measuring the individual tracks of 20 randomly selected individual cells per experimental condition, where each condition in an experiment was repeated at least three times. Flow cytometry assay quantification was based on triplicate samples in each experiment from three independent experiments as percentage (%). The data are presented as mean ± s.d. Statistical differences were evaluated using the two-tailed Student t-test for comparisons of two groups, or analysis of variance for comparisons of more than two groups. *p* < 0.05 was considered significant.

## Additional Information

**How to cite this article**: Dong, H. *et al.* Breast Cancer MDA-MB-231 Cells Use Secreted Heat Shock Protein-90alpha (Hsp90α) to Survive a Hostile Hypoxic Environment. *Sci. Rep.* **6**, 20605; doi: 10.1038/srep20605 (2016).

## Supplementary Material

Supplementary Information
