# Supplementary material for: Breast Cancer MDA-MB-231 Cells Use Secreted Heat Shock Protein-90alpha (Hsp90α) to Survive a Hostile Hypoxic Environment
Source: Sci Rep. 2016 Feb 5;6:20605. doi: 10.1038/srep20605 (PMC4742873; doi:10.1038/srep20605)
Supplement: Supplementary Information [file srep20605-s1.pdf]

Breast Cancer MDA-MB-231 Cells Use Secreted Heat Shock Protein-90alpha (Hsp90α) to Survive a Hostile Hypoxic Environment

Hangming Dong, Mengchen Zou, Ayesha Bhatia, Priyamvada Jayaprakash, Florence Hofman, Qilong Ying, Mei Chen, David T. Woodley and Wei Li

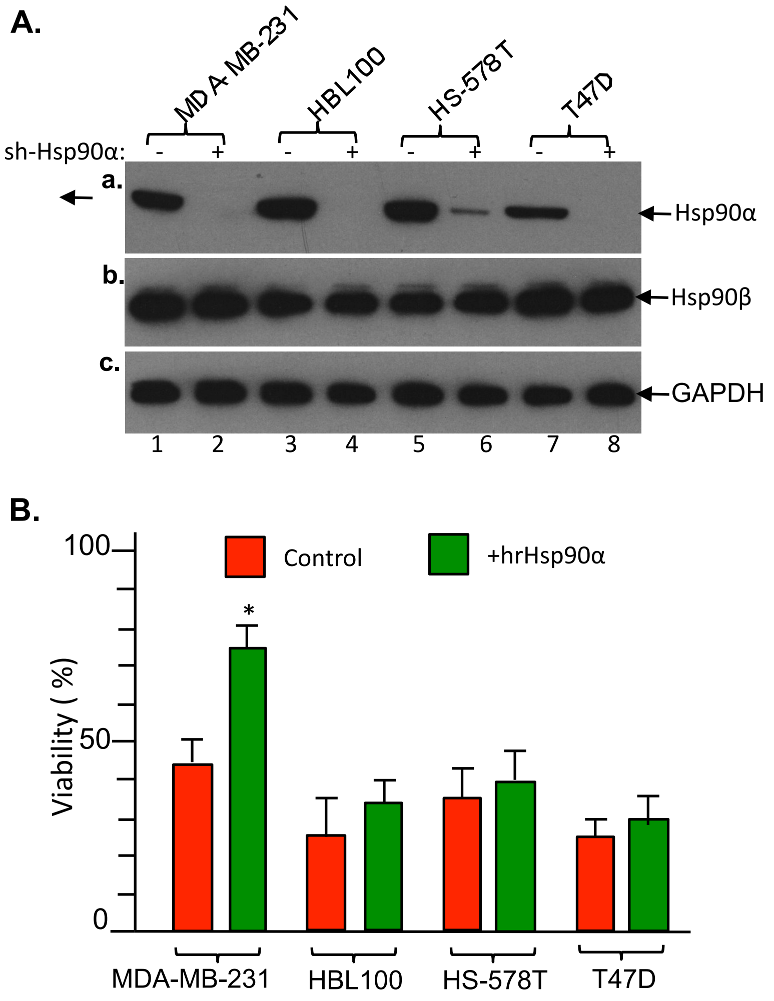

Figure 1s. Rescue of various Hsp90α-knockout breast cancer cells
(A) Down-regulation of Hsp90α as shown by Western immune blotting with anti-Hsp90α or Hsp90β antibody.
(B) Hsp90α-knockdown cells were subjected hypoxia (1% oxygen, 48 hours) in the absence or presence of added recombinant Hsp90α. Cell viability was measured by flow cytometry as quantitated as % of viable cells from three experiments. * $p < 0.05$.
